# Risk Factors for Anxiety or Depression in Patients With Stroke During the Acute and Recovery Phases: An Independent Cohort Study

**DOI:** 10.62641/aep.v54i4.2286

**Published:** 2026-08-15

**Authors:** Mei Li, Chuchu Ge, Yu Han, Yan Tang, Li Zhao

**Affiliations:** ^1^Department of Neurology, Nantong Rici Hospital Affiliated to Yangzhou University, 226010 Nantong, Jiangsu, China

**Keywords:** cerebrovascular diseases, elderly, anxiety, depression, risk factors

## Abstract

**Background:**

To identify the overall risk factors for any clinically significant anxiety or depressive anxiety or depression in patients with stroke in the acute and recovery phase cohorts, compare the differences in overall risk factors between two independent patient cohorts at different disease stages, so as to provide a reliable basis for early screening and staged intervention of post-stroke emotional anxiety or depression.

**Methods:**

This is a retrospective study that included 252 stroke patients admitted to Nantong Rici Hospital Affiliated to Yangzhou University from August 2023 to October 2025, which were divided into two independent cohorts: the acute phase cohort (7–14 days after onset, n = 143) and the recovery phase cohort (3 months after onset, n = 109), all eligible patients were assigned to either the anxiety or depression group or the non-anxiety or depression group according to the 14-item Hamilton Anxiety Rating Scale (HAMA-14) and 24-item Hamilton Depression Rating Scale (HAMD-24). Following univariate analysis-based screening of potential risk factors, the significantly different indicators were further included in the multivariate Logistic regression model to identify independent determinants and clarify differences across stages.

**Results:**

In the acute phase cohort, the incidence of anxiety or depression was 30.07% (43/143), and the independent risk factors identified were age [odds ratio (OR) = 1.23, 95% confidence interval (CI): 1.10–1.38], illiterate educational level (OR = 8.15, 95% CI: 2.28–29.12), no dedicated care (OR = 5.52, 95% CI: 1.58–19.34), hemorrhagic stroke (OR = 6.39, 95% CI: 1.61–25.30), multiple lesions (OR = 5.07, 95% CI: 1.31–19.66), National Institutes of Health Stroke Scale (NIHSS) score (OR = 1.25, 95% CI: 1.03–1.52), and combined atrial fibrillation (AF) (OR = 5.38, 95% CI: 1.20–24.09) (*p* all < 0.05). In the recovery phase cohort, the incidence of anxiety or depression was 25.69% (28/109), and the independent risk factors identified were age (OR = 1.21, 95% CI: 1.05–1.39), combined diabetes mellitus (DM) (OR = 4.63, 95% CI: 1.18–18.20), AF (OR = 3.94, 95% CI: 1.07–14.45), and coronary heart disease (CHD) (OR = 5.86, 95% CI: 1.45–23.73) (*p* all < 0.05). Age and combined AF were the common independent risk factors in both cohorts.

**Conclusions:**

There are differences in the risk factors for anxiety/depression between independent stroke patients cohorts at different disease stages. Clinically, individualized intervention should be carried out according to the key risk factors of each disease stage to reduce the risk of anxiety or depression.

## Introduction

As the population ages, cerebrovascular diseases (CVD, hereinafter referred to as CVD to avoid confusion with cardiovascular disease) show a rising incidence among the elderly, representing a major contributor to global disability and mortality [1]. Stroke, including ischemic stroke and hemorrhagic stroke, is the most common clinical subtype of CVD, while cardiovascular disease refers to a group of anxiety or depression affecting the heart and blood vessels (e.g., coronary heart disease (CHD), atrial fibrillation), which are important comorbidities of stroke [2]. Patients with stroke, due to brain tissue injury, neurological deficits, and long-term rehabilitation pressure, often suffer from anxiety or depression such as anxiety and depression, reducing their quality of life while possibly delaying neurological function recovery and increasing disability and mortality rates [3, 4]. According to statistics, about 30%–50% of stroke patients develop emotional anxiety or depression in the acute stage (within one month of onset) [5], and the prevalence further increases to 40%–60% during the recovery phase cohort (from one to six months after onset) [6], becoming a key independent factor affecting prognosis. Age, sex, and past history of mental illness are established common risk factors [7, 8]. Furthermore, there is increasing evidence that the progression of stroke is closely related to anxiety and depression, creating a vicious cycle that exacerbates both neurological and psychiatric outcomes [6, 9]. However, the existing studies focus on analyzing risk factors in a single disease stage [10, 11], and lack comparative studies on risk factors between independent patient populations in the acute and recovery phase cohort. Moreover, the evidence is largely obtained from multi-center and large sample survey [12], which cannot comprehensively reflect the clinical characteristics of patients in specific regions and medical institutions. Furthermore, for the population, a special group, the differences and correlations of risk factors at different disease stages have not been fully clarified, and the related conclusions remain controversial.

In light of this, the present study, based on the single-center clinical data, conducted a comparative study on the risk factors of anxiety or depression in two independent cohorts of stroke patients at the acute and recovery phase cohort, and clarified the key risk factors for each phase cohort. The findings are expected to lay a reliable foundation for the clinical formulation of phase cohort and individualized prevention and intervention strategies, so as to better identify high-risk groups to allow for timely intervention measures and thus reduce the incidence of anxiety/depression, ultimately enhancing patients’ rehabilitation outcomes and quality of life.

## Materials and Methods

### Research Participants

A total of 252 patients with stroke admitted to Nantong Rici Hospital Affiliated to Yangzhou University from August 2023 to October 2025 were enrolled for retrospective analysis: the acute phase cohort (7–14 days after onset, n = 143) and the recovery phase cohort (3 months after onset, n = 109). All patients were diagnosed with stroke (ischemic/hemorrhagic) by cranial computed tomography (CT) or magnetic resonance imaging (MRI). Ischemic stroke was diagnosed according to the Chinese Guidelines for the Diagnosis and Treatment of Acute Ischemic Stroke 2023, defined as acute focal neurological dysfunction caused by cerebral infarction confirmed by diffusion-weighted imaging (DWI) or cranial CT. Hemorrhagic stroke was diagnosed based on the Chinese Guidelines for the Diagnosis and Treatment of Intracerebral Hemorrhage 2023, confirmed by the presence of acute intracerebral hemorrhage on cranial CT or MRI [13].

A total of 276 consecutive stroke patients admitted to our hospital between August 2023 and October 2025 were initially assessed for eligibility. Among them, 24 were excluded: 11 due to previous or family history of mental illness, 7 due to severe comorbidities (hepatorenal insufficiency, malignant tumor, serious infection), and 6 due to severe cognitive dysfunction that prevented valid psychological assessment. Ultimately, 252 eligible patients were enrolled in the final analysis, including 143 in the acute phase cohort and 109 in the recovery phase cohort. The Ethics Committee of Nantong Rici Hospital Affiliated to Yangzhou University has granted approval for this research project (No. NTRC-LL2026-032). Anonymized data collection was implemented, and all participants provided written informed consent after being clearly informed of the study objectives, methods, and potential risks. The two cohorts were independent of each other with no overlapping patients. The acute phase cohort included patients admitted within 7–14 days of stroke onset for acute treatment, while the recovery phase cohort included patients admitted 3 months after onset for standardized rehabilitation.

### Case Selection Criteria

Eligibility criteria: (1) Age ≥ 18 years; (2) The initial diagnosis was stroke; (3) The condition was in the acute phase (7–14 days after onset) or recovery phase (3 months after onset) at the time of psychological assessment, which falls within the standard time windows defined by guidelines for stroke [14]; (4) Complete clinical data, including demographic information, imaging findings, neurological function evaluation records, mental and psychological state evaluation data, etc. Ineligibility criteria: (1) Previous history of mental illness (anxiety, depression, schizophrenia, etc.) or family mental illness history; (2) Severe hepatorenal insufficiency, malignant tumor, serious infection, or other major physical diseases; (3) Serious cognitive dysfunction hindering psychological assessments.

### Data Extraction

A unified clinical data collection table was designed, and two systematically trained researchers independently extracted the following data from the hospital electronic medical record system: (1) Demographic characteristics: age, sex, educational level (illiterate = no formal education; non-illiterate = primary school education or above), and family support (no dedicated care = no full-time caregiver; dedicated care = having a full-time caregiver); (2) Disease characteristics: stroke type (ischemic/hemorrhagic), number of lesions (single/multiple), admission National Institutes of Health Stroke Scale (NIHSS) score, and comorbidities (diabetes mellitus (DM), atrial fibrillation, CHD, hypertension); (3) Biochemical indicators: homocysteine, total cholesterol, triglyceride, low-density lipoprotein cholesterol, high-density lipoprotein cholesterol; (4) Psychological assessment results: 14-item Hamilton Anxiety Rating Scale (HAMA-14) and 24-item Hamilton Depression Rating Scale (HAMD-24) scores. Disagreements during data extraction were resolved by a third-party senior neurologist.

### Outcomes

Assessment data during the acute (7–14 d after admission) and recovery (3 months after onset) stages were retrospectively collected from medical records, all of which were generated using standardized scales as follows:

(1) Anxiety symptoms were assessed using the HAMA-14, originally developed by Hamilton [15]. This scale has been validated in Chinese patients [16] and shows good psychometric properties (Cronbach’s α = 0.85). It consists of 14 items, divided into two dimensions: Psychic Anxiety (7 items: anxious mood, tension, fears, insomnia, cognitive disturbance, depressed mood, behavior during interview) and Somatic Anxiety (7 items: muscular system symptoms, sensory system symptoms, cardiovascular system symptoms, respiratory system symptoms, gastrointestinal system symptoms, genitourinary system symptoms, autonomic nervous system symptoms). Each item was rated on a 5-point scale (0 = no symptom, 1 = mild, 2 = moderate, 3 = severe, 4 = extremely severe) by trained neurologists through face-to-face interviews at the corresponding time points, as documented in medical records. A total score of ≥14 was used to define clinically significant anxiety symptoms, consistent with previous studies in Chinese stroke patients [17].

(2) Depression status: Depressive symptoms were assessed using the HAMD-24, an expanded clinician-rated version of the Hamilton depression scale [18]. Previous studies in post-stroke patients have used HAMD-24 to evaluate depressive symptom severity [16, 19]. The HAMD-24, an expanded form of the Hamilton Depression Rating Scale originally developed by Hamilton, was used to assess depressive symptom severity. The scale comprises 24 clinician-rated items, including depressed mood, guilt, suicide, three insomnia items, work and activities, retardation, agitation, psychic and somatic anxiety, gastrointestinal and general somatic symptoms, genital symptoms, hypochondriasis, weight loss, insight, diurnal variation, depersonalization/derealization, paranoid symptoms, obsessive-compulsive symptoms, helplessness, hopelessness, and worthlessness. Most items are scored from 0 to 4, whereas several items are scored from 0 to 2, yielding a total score range of 0–76. In the present study, HAMD-24 ≥ 20 was used to define clinically significant depressive symptoms, based on previously reported HAMD-24 severity categories [19].

(3) Neurological function deficit: Assessed using the NIHSS [20], which has been validated in Chinese stroke patients with good psychometric properties (Cronbach’s α = 0.84) [21]. The scale adopts a fixed severity-based rating system: most items are scored on a 0–2 or 0–3 scale, while upper and lower limb motor items use a 0–4 scale (0 = no neurological deficit, higher scores indicate more severe impairment), with a total score ranging from 0 to 42. All evaluations were independently completed by two systematically trained neurologists and documented in medical records; any inter-rater difference exceeding 2 points prompted a final review and confirmation by a third-party senior physician.

Based on the HAMA-14 and HAMD-24 scores extracted from medical records, patients in the acute and recovery phase cohorts were separately assigned to two groups: (a) Patients with HAMA-14 ≥ 14 and/or HAMD-24 ≥ 20 were assigned to the clinically significant anxiety or depressive symptoms group [17, 19]; (b) Non-anxiety or depression group (preset as the reference group, defined as HAMA-14 < 14 and HAMD-24 < 20, with no clinically significant anxiety or depressive symptoms).

### Statistical Analyses

SPSS 26.0 (IBM, Armonk, NY, USA) was used for all statistical analyses. All continuous variables in this study followed a normal distribution (verified by Shapiro–Wilk test), so they were expressed as mean ± standard deviation ($\bar{x}$ ± s), and between-group differences were examined using independent sample *t*-tests. Categorical variables were expressed as n (%), and between-group differences were examined using chi-square tests. Variables with *p* < 0.050 in univariate analysis were further included in multivariate binary logistic regression models (conducted separately for each cohort) to identify independent risk factors. All tests were two-sided, and a *p*-value < 0.050 was considered statistically significant.

## Results

### Baseline Data of Research Participants

This study included 139 males and 113 females aged 53–89 (mean: 72.63 ± 7.08) years. Among them, 7 cases (2.78%) in the 50–59 age years old, 77 cases (30.56%) were 60–69 years old, 124 cases (49.21%) were in the 70–79 age group, and 44 cases (17.46%) were aged over 80. Ischemic and hemorrhagic strokes were diagnosed in 131 (51.98%) and 121 (48.02%), respectively. Among them, 71 cases (28.17%) of the patients had anxiety or depression. In the acute phase cohort, the incidence of anxiety or depression was 30.07% (43/143). In the recovery phase cohort, the incidence of anxiety or depression was 25.69% (28/109) (Table 1).

**Table 1.** **Baseline characteristics of patients with stroke**.

Variable		n	%
Male		139	55.16
Female		113	44.84
Age ($\bar{x}$ ± s)		72.63 ± 7.08	
Age (min–max)		53–89	
Age (percentage)			
	50–59	7	2.78
	60–69	77	30.56
	70–79	124	49.21
	≥ 80	44	17.46
Stroke type			
	Hemorrhagic	121	48.02
	Ischemic	131	51.98
Study cohort			
	Acute phase cohort	143	56.75
	Recovery phase cohort	109	43.25
Mental and psychological			
	Anxiety or depression	71	28.17
	Non-anxiety or depression	181	71.83
HAMA-14		14.30 ± 7.49	
HAMD-24		17.86 ± 9.63	

Abbreviations: HAMA-14, 14-item Hamilton Anxiety Rating Scale; HAMD-24, 24-item Hamilton Depression Rating Scale.

### Univariate Analysis of Anxiety or Depression in the Acute Stage

For the patients in the acute stage, we conducted a comparative analysis of various endpoints between the anxiety or depression group (43 cases) and the non-anxiety or depression group (100 cases). Age, illiterate educational level, no dedicated caregiver, hemorrhagic stroke, multiple lesions, NIHSS score, combined with DM/atrial fibrillation (AF), homocysteine (Hcy) is a risk factor for acute-phase cohort anxiety or depression (*p* < 0.05). Sex, combined hypertension (HTN)/CHD, and blood lipid-related indexes (TC, TG, LDL-C, HDL-C) showed no marked between-group differences (*p* > 0.05) (Table 2).

**Table 2.** **Univariate analysis of anxiety or depression in the acute phase cohort of stroke patients**.

Variable		Non-anxiety and non-depression (n = 100)	Anxiety or depression (n = 43)	*t* or χ^2^	*p*
Age		70.78 ± 7.36	77.40 ± 5.02	5.375	<0.001
Sex				0.752	0.386
	Male	59 (59.00)	22 (51.16)		
	Female	41 (41.00)	21 (48.84)		
Educational level				11.861	<0.001
	Illiterate	34 (34.00)	28 (65.12)		
	Non-illiterate	66 (66.00)	15 (34.88)		
Family support				13.341	<0.001
	No dedicated care	46 (46.00)	34 (79.07)		
	Dedicated care	54 (54.00)	9 (20.93)		
Stroke type				7.786	0.005
	Hemorrhagic	42 (42.00)	29 (67.44)		
	Ischemic	58 (58.00)	14 (32.56)		
Number of lesions				5.230	0.022
	Single	77 (77.00)	25 (58.14)		
	Multiple	23 (23.00)	18 (41.86)		
NIHSS		12.598 ± 3.95	15.77 ± 3.07	4.698	<0.001
Combined DM				4.263	0.039
	Yes	35 (35.00)	23 (53.49)		
	No	65 (65.00)	20 (46.51)		
Combined AF				3.909	0.048
	Yes	14 (14.00)	12 (27.91)		
	No	86 (86.00)	31 (72.09)		
Combined CHD				1.064	0.302
	Yes	16 (16.00)	10 (23.26)		
	No	84 (84.00)	33 (76.74)		
Combined HTN				1.519	0.218
	Yes	54 (54.00)	28 (65.12)		
	No	46 (46.00)	15 (34.88)		
Hcy (μmol/L)		10.19 ± 4.97	13.90 ± 4.29	4.257	<0.001
TC (mmol/L)		5.59 ± 1.31	5.84 ± 2.02	0.888	0.376
TG (mmol/L)		1.69 ± 0.22	1.77 ± 0.34	1.765	0.080
LDL-C (mmol/L)		3.74 ± 0.44	3.87 ± 0.55	1.520	0.131
HDL-C (mmol/L)		1.03 ± 0.22	0.98 ± 0.09	1.587	0.115

Abbreviations: NIHSS, National Institutes of Health Stroke Scale; DM, diabetes mellitus; AF, atrial fibrillation; CHD, coronary heart disease; HTN, hypertension; Hcy, homocysteine; TC, total cholesterol; TG, triglyceride; LDL-C, low-density lipoprotein cholesterol; HDL-C, high-density lipoprotein cholesterol.

### Multivariate Analysis of Anxiety or Depression During the Acute Phase Cohort

9 indexes screened out by univariate analysis (*p* < 0.05) were included in the multivariate Logistic regression model (the assignment situation is shown in Table 3). The results showed that age [odds ratio (OR) = 1.23, 95% confidence interval (CI): 1.10–1.38], educational level (illiterate) (OR = 8.15, 95% CI: 2.28–29.12), absence of dedicated care (OR = 5.52, 95% CI: 1.58–19.34), hemorrhagic stroke (OR = 6.39, 95% CI: 1.61–25.30), number of lesions (OR = 5.07, 95% CI: 1.31–19.66) NIHSS score (OR = 1.25, 95% CI: 1.03–1.52), , and combined AF (OR = 5.38, 95% CI: 1.20–24.09) are independent risk factors for anxiety or depression in patients with stroke during the acute phase cohort (*p* all < 0.05). However, the coexistence of DM and Hcy has no effect on anxiety or depression (*p* all > 0.05) (Table 4).

**Table 3.** **Assignment of variables in the multivariate logistic regression model (acute phase cohort)**.

Variable		Assignment
Dependent variable		
	Anxiety or depression	Non-anxiety or depression = 1, anxiety or depression = 2
Covariate		
	Age	Use the original data
	Educational level	Non-illiterate = 1, illiterate = 2
	Family support	Dedicated care = 1, no dedicated care = 2
	Stroke type	Ischemic = 1, hemorrhagic = 2
	Number of lesions	Single = 1, multiple = 2
	Combined DM/AF	No = 1, yes = 2
	Hcy (μmol/L)	Use the original data
	NIHSS	Use the original data

Abbreviations: DM, diabetes mellitus; AF, atrial fibrillation; Hcy, homocysteine; NIHSS, National Institutes of Health Stroke Scale.

**Table 4.** **Multivariate logistic regression analysis of anxiety or depression in the acute phase cohort of stroke patients**.

Variable	B	SE	Wald χ^2^	*p*	OR	95% CI
Age	0.21	0.06	12.219	<0.001	1.23	1.10–1.38
Educational level	2.10	0.65	10.439	0.001	8.15	2.28–29.12
Family support	1.71	0.64	7.136	0.008	5.52	1.58–19.34
Stroke type	1.86	0.70	6.980	0.008	6.39	1.61–25.30
Number of lesions	1.62	0.69	5.498	0.019	5.07	1.31–19.66
NIHSS	0.22	0.10	4.918	0.027	1.25	1.03–1.52
Combined DM	1.18	0.60	3.844	0.050	3.25	1.00–10.53
Combined AF	1.68	0.76	4.849	0.028	5.38	1.20–24.09
Hcy	0.10	0.06	2.557	0.110	1.10	0.98–1.25

Abbreviations: B, regression coefficient; SE, standard error; OR, odds ratio; CI, confidence interval; DM, diabetes mellitus; AF, atrial fibrillation; Hcy, homocysteine; NIHSS, National Institutes of Health Stroke Scale.

### Univariate Analysis of Anxiety or Depression in the Recovery Period

The outcome measures of the anxiety or depression (28 cases) and non-anxiety and non-depression (81 cases) groups in the recovery period were comparatively analyzed. Age, educational level (illiterate), lack of dedicated care, NIHSS, combined with DM/AF, and Hcy are all risk factors for anxiety or depression during the recovery period (*p* < 0.05); however, unlike the baseline period, the number of people with CHD/HTN in the recovery period anxiety or depression group was also higher (*p* < 0.05). In contrast, the two cohorts differed non-significantly in the stroke type, lesion number, and blood lipid-related indices (*p* > 0.05) (Table 5).

**Table 5.** **Univariate analysis of anxiety or depression in the recovery phase cohort of stroke patients**.

Variable		Non-anxiety and non-depression (n = 81)	Anxiety or depression (n = 28)	*t* or χ^2^	*p*
Age		71.31 ± 6.65	75.75 ± 5.78	3.146	0.002
Sex				0.234	0.629
	Male	42 (51.85)	16 (57.14)		
	Female	39 (48.15)	12 (42.86)		
Educational level				7.534	0.006
	Illiterate	28 (34.57)	18 (64.29)		
	Non-illiterate	53 (65.43)	10 (35.71)		
Family support				6.628	0.010
	No dedicated care	46 (56.79)	8 (28.57)		
	Dedicated care	35 (43.21)	20 (71.43)		
Stroke type				1.928	0.165
	Hemorrhagic	34 (41.98)	16 (57.14)		
	Ischemic	47 (58.02)	12 (42.86)		
Number of lesions				1.061	0.303
	Single	55 (67.90)	16 (57.14)		
	Multiple	26 (32.10)	12 (42.86)		
NIHSS		13.72 ± 3.83	16.96 ± 3.39	3.979	<0.001
Combined DM				11.473	<0.001
	Yes	28 (34.57)	20 (71.43)		
	No	53 (65.43)	8 (28.57)		
Combined AF				10.202	0.001
	Yes	22 (27.16)	17 (60.71)		
	No	59 (72.84)	11 (32.29)		
Combined CHD				9.906	0.002
	Yes	20 (24.69)	16 (57.14)		
	No	61 (75.31)	12 (42.86)		
Combined HTN				7.534	0.006
	Yes	28 (34.57)	18 (64.29)		
	No	53 (65.43)	10 (35.71)		
Hcy (μmol/L)		12.87 ± 3.60	14.91 ± 4.96	2.340	0.021
TC (mmol/L)		5.19 ± 0.61	5.29 ± 0.56	0.845	0.400
TG (mmol/L)		2.01 ± 0.31	2.15 ± 0.53	1.604	0.112
LDL-C (mmol/L)		3.74 ± 0.41	3.91 ± 0.23	1.955	0.053
HDL-C (mmol/L)		1.06 ± 0.23	0.99 ± 0.14	1.371	0.173

Abbreviations: NIHSS, National Institutes of Health Stroke Scale; DM, diabetes mellitus; AF, atrial fibrillation; CHD, coronary heart disease; HTN, hypertension; Hcy, homocysteine; TC, total cholesterol; TG, triglyceride; LDL-C, low-density lipoprotein cholesterol; HDL-C, high-density lipoprotein cholesterol; SD, standard deviation.

### Multivariate Analysis of Anxiety or Depression in the Recovery Phase Cohort

The 9 univariate analysis-identified significant factors (*p* < 0.05) were incorporated into the multivariate Logistic regression model (Table 6). Age (OR = 1.21, 95% CI: 1.05–1.39), combined DM (OR = 4.63, 95% CI: 1.18–18.20), combined AF (OR = 3.94, 95% CI: 1.07–14.45), CHD (OR = 5.86, 95% CI: 1.45–23.73) were confirmed as independent risk factors for anxiety or depression in patients with stroke in the recovery period (*p* all < 0.05). Educational level, family support, NIHSS, combined HTN, and Hcy were all excluded (*p* all > 0.05) (Table 7).

**Table 6.** **Assignment of variables in the multivariate logistic regression model (recovery phase cohort)**.

Variable		Assignment
Dependent variable		
	Anxiety or depression	Non-anxiety or depression = 1, anxiety or depression = 2
Covariate		
	Age	Use the original data
	Educational level	Non-illiterate = 1, illiterate = 2
	Family support	Dedicated care = 1, no dedicated care = 2
	Combined DM/AF/CHD/HTN	No = 1, yes = 2
	NIHSS	Use the original data
	Hcy	Use the original data

Abbreviations: DM, diabetes mellitus; AF, atrial fibrillation; CHD, coronary heart disease; HTN, hypertension; NIHSS, National Institutes of Health Stroke Scale; Hcy, homocysteine.

**Table 7.** **Multivariate logistic regression analysis of anxiety or depression in the recovery phase cohort of stroke patients**.

Variable	B	SE	Wald χ^2^	*p*	OR	95% CI
Age	0.19	0.07	6.87	0.01	1.21	1.05–1.39
Educational level	1.04	0.66	2.43	0.12	2.82	0.77–10.35
Family support	–1.18	0.68	3.00	0.08	0.31	0.08–1.17
NIHSS	0.18	0.10	3.42	0.07	1.20	0.99–1.45
Combined DM	1.53	0.70	4.82	0.03	4.63	1.18–18.20
Combined AF	1.37	0.66	4.26	0.04	3.94	1.07–14.45
Combined CHD	1.77	0.71	6.14	0.01	5.86	1.45–23.73
Combined HTN	0.93	0.68	1.86	0.17	2.54	0.67–9.72
Hcy	0.09	0.08	1.17	0.28	1.09	0.93–1.28

Abbreviations: B, regression coefficient; SE, standard error; OR, odds ratio; CI, confidence interval; DM, diabetes mellitus; AF, atrial fibrillation; CHD, coronary heart disease; HTN, hypertension; Hcy, homocysteine; NIHSS, National Institutes of Health Stroke Scale.

### Differences in Risk Factors Between the Acute and Recovery Phase Cohort

By comparing the analysis results of risk factors for anxiety or depression between the acute and recovery phase cohort, the following characteristics were identified: (a) Common risk factors: age and combined AF were independent risk factors for anxiety/depression in both cohorts, with both having a strong predictive effect (OR > 2.0); (b) Acute phase cohort-specific risk factors: educational level, family support, stroke type, number of lesions, and NIHSS score were independent risk factors unique to the acute phase cohort, which were not statistically significant in the recovery phase cohort; (c) Recovery phase cohort-specific risk factors: combined DM and combined CHD were independent risk factors unique to the recovery phase cohort. Although combined DM was identified as a risk factor in the univariate analysis of the acute phase cohort, it did not enter the multivariate regression model (Table 8).

**Table 8.** **Differences in risk factors for anxiety or depression between the acute and recovery phase cohort**.

Type	Acute phase cohort	Recovery phase cohort
Unique	Educational level, family support, type of stroke, number of lesions, NIHSS	Combined DM/CHD
Co-owned	Age, combined AF	

Abbreviations: NIHSS, National Institutes of Health Stroke Scale; DM, diabetes mellitus; AF, atrial fibrillation; CHD, coronary heart disease.

## Discussion

This study focused on the overall risk of any clinically significant emotional anxiety or depression (including pure anxiety, pure depression, and comorbid anxiety and depression) after stroke, and clarified the similarities and differences of the overall risk factors between the acute and recovery phases of stroke, which can provide a simple and practical reference for clinical early screening of high-risk populations prone to post-stroke emotional anxiety or depression. The results showed that the incidence of anxiety/depression in the acute phase cohort and recovery phase cohort was 30.07% (43/143) and 25.69% (28/109), respectively. Age and combined AF were the common independent risk factors in both cohorts; educational level, family support, stroke type, number of lesions, and NIHSS score were the unique independent risk factors in the acute phase cohort, while combined DM and combined CHD were the unique independent risk factors in the recovery phase cohort. Our findings provide a theoretical basis for phased clinical intervention for stroke patients with anxiety/depression.

Age ≥ 80 years was identified as an independent risk factor for anxiety and depression during both the acute and recovery phase cohort of stroke, challenging prior assumptions regarding stage-specific effects and more accurately reflecting the physiological and psychological vulnerabilities of patients. From a pathophysiological standpoint, individuals aged ≥ 80 years exhibit widespread cerebral degenerative changes, including reduced neuronal density, impaired synaptic connectivity, and significantly diminished functional reserve in emotion-regulating regions such as the prefrontal cortex and limbic system [22]. During the acute phase cohort, stroke-related insults—such as ischemia- or hemorrhage-induced brain edema and neuronal necrosis—superimpose directly on this compromised neural substrate, resulting in dual-pathway disruption of emotional regulation. First, acute lesions amplify neuroinflammatory responses and exacerbate dysregulation of key neurotransmitters, including serotonin and dopamine [23]. Second, older adults demonstrate limited resilience to acute physiological stressors; sudden impairments in motor function or loss of independence often precipitate rapid-onset feelings of helplessness and despair. Critically, these affective disturbances are not subsumed by somatic symptoms but instead emerge as primary drivers of anxiety and depressive anxiety or depression [24]. In the recovery phase cohort, the influence of advanced age becomes increasingly evident. Despite resolution of acute neurological deficits, patients face inherently limited neuroplasticity and reduced rehabilitation capacity, leading to protracted recovery of motor, linguistic, and cognitive functions. Persistent functional impairments thus serve as enduring chronic stressors [25]. Moreover, structural weaknesses in social support systems—including inadequate caregiving resources and restricted social networks—limit access to emotional reinforcement and motivational encouragement. This deficit facilitates the progressive accumulation of negative affect, thereby sustaining or worsening anxiety and depression over time. Collectively, these findings underscore that advanced age constitutes a persistent, phase cohort-independent risk factor requiring targeted clinical monitoring throughout the disease trajectory.

The presence of AF was also confirmed as an independent predictor of anxiety and depression across both phase cohort, addressing previous underestimation of its psychosomatic impact and highlighting the bidirectional interplay between cardiovascular comorbidities and mental health outcomes. Pathophysiologically, the co-occurrence of AF and stroke exerts synergistic detrimental effects on brain-heart axis integrity. In the acute phase cohort, patients confront combined cerebral injury and emotional anxiety or depression, compounding systemic instability [26]. Furthermore, AF is associated with elevated risks of thromboembolic events and clinical deterioration, fostering profound uncertainty about prognosis. This perceived unpredictability induces significant psychological distress, directly contributing to the onset of depressive symptomatology—an effect that remains robust after adjusting for stroke severity (e.g., NIHSS score) and stroke subtype, affirming its independence as a risk factor [27]. During recovery, the adverse influence of AF persists through two interconnected mechanisms: “chronic risk apprehension” and “rehabilitation interference.” First, as a lifelong arrhythmia, AF necessitates long-term anticoagulation therapy (e.g., warfarin or novel oral anticoagulants), which may provoke sustained anxiety due to concerns about bleeding complications and the burden of routine coagulation monitoring [28]. Second, reduced exercise tolerance in AF patients constrains the intensity and consistency of rehabilitative interventions, undermining functional gains and fostering a sense of therapeutic futility, which further exacerbates depressive symptoms [29]. Additionally, the substantially increased risk of recurrent stroke in AF patients perpetuates a state of chronic psychological vigilance; persistent fear of relapse impedes the development of confidence in recovery, thereby maintaining maladaptive emotional states. These dynamics illustrate how AF extends beyond cardiovascular morbidity to exert enduring effects on mental well-being across the entire care continuum.

Hemorrhagic stroke is an independent risk factor specific to the acute stage. This is because such a stroke type occurs more rapidly and has a more severe condition. The mechanical compression of brain tissue caused by intracerebral hemorrhage is more severe, not only exacerbating neurological function damage, but also triggering neurochemical imbalances through the stress axis, leading to a sharp increase in anxiety and depression [30]. In contrast, ischemic stroke is relatively mild in terms of its condition, and the psychological adaptation period for patients is longer [31]. Therefore, it does not become an independent risk factor. The mechanism underlying the effect of Hcy may be related to the damage of the vascular endothelium by high Hcy through oxidative stress and the aggravation of cerebral ischemia and hypoxia [32]. Meanwhile, it can affect the synthesis and metabolism of mood-related neurotransmitters such as serotonin and dopamine, further inducing psychological anxiety or depression on the basis of acute brain tissue injury [33]. However, the influence of Hcy was weakened in the recovery phase cohort as the condition stabilized and treatment interventions reduced Hcy levels, which is consistent with the relevant clinical research results of Tao *et al*. [34].

Based on these findings, we hold that clinical interventions should be carried out in stages when dealing with patients suffering from stroke. For instance, during the acute phase cohort, patients with hemorrhagic stroke, high Hcy levels, and high NIHSS scores should be closely monitored. While emphasizing prompt condition control, folic acid supplementation for Hcy level down-regulation and early psychological counseling should be provided. For the recovery phase cohort, special attention should be paid to patients with advanced age, combined diabetes, and no dedicated care. It is important to strictly control blood sugar while strengthening rehabilitation training guidance and improving the care support system. However, this study is a retrospective single-center study, with limitations such as limited sample representativeness and potential selection bias. In particular, the recovery phase cohort only included patients hospitalized for rehabilitation, which may overestimate the prevalence of anxiety or depression as patients with more severe functional impairment are more likely to receive inpatient rehabilitation. These findings should be generalized with caution to other regions and medical institutions. Additionally, the relatively small single-center sample size limits the statistical power of subgroup analyses, and thus the conclusions should be interpreted with caution. Secondly, as a retrospective study that only analyzes independent cohorts at different disease stages, it is impossible to clarify the dynamic prognosis pattern and long-term influencing factors of psychological anxiety or depression during the progression of stroke. Meanwhile, this study focused on the overall risk factors for post-stroke emotional anxiety or depression, and did not further analyze the phenotype-specific risk factors for pure anxiety, pure depression and anxiety-depression comorbidity due to the limited sample size of single-center research. Subsequent multi-center studies with larger sample sizes can further explore the specific influencing factors of different emotional anxiety or depression phenotypes, so as to obtain more precise intervention targets for subtype-specific treatment.

## Conclusions

This single-center retrospective study preliminarily clarified the common and unique independent risk factors for anxiety/depression in two independent cohorts of stroke patients at the acute and recovery phases, and revealed the stage-specific characteristics of these risk factors. The findings provide a reliable basis for clinical formulation of phase and individualized prevention and intervention strategies, which is helpful for early identification of high-risk groups and timely adoption of targeted measures to reduce the incidence of anxiety/depression, ultimately contributing to enhanced rehabilitation effects and quality of life for patients.

## Availability of Data and Materials

All experimental data included in this study can be obtained by contacting the first author if needed.

## References

[1] Cui X, Zhang Y, Jin Y, Wu Z, Gao Y, Su H (2025). Association of cardiometabolic index and depressive symptoms with cardiovascular and cerebrovascular diseases in middle-aged and older adults: A national prospective cohort study. *Journal of affective disorders*.

[2] Pannell JS, Corey AS, Shih RY, Austin MJ, Chu S, Expert Panel on Neurological Imaging (2024). ACR Appropriateness Criteria^®^ Cerebrovascular Diseases-Stroke and Stroke-Related Conditions. *Journal of the American College of Radiology : JACR*.

[3] Lao P, Young CB, Ezeh C, Lacayo B, Seblova D, Andrews RM (2024). Loneliness, cerebrovascular and Alzheimer’s disease pathology, and cognition. Alzheimer’s & dementia : the journal of the Alzheimer’s Association. *Alzheimer’s & dementia : the journal of the Alzheimer’s Association*.

[4] Wang S, Yin X, Jiang T, Xu J, Wang D (2024). Impact of Cardiovascular and Cerebrovascular Diseases Mortality on Life Expectancy in Tianjin, 2004 and 2020. *Asia-Pacific journal of public health*.

[5] Zhu Y, Que D, Jin Z, Zhang X, Song X, Chen K (2025). Association of different emotional support status with cardio-cerebrovascular diseases. *Journal of affective disorders*.

[6] Gu P, Ding Y, Ruchi M, Feng J, Fan H, Fayyaz A (2024). Post-stroke dizziness, depression and anxiety. *Neurological research*.

[7] Calatayud E, Marcen-Roman Y, Rodriguez-Roca B, Salavera C, Gasch-Gallen A, Gomez-Soria I (2023). Sex differences on anxiety and depression in older adults and their relationship with cognitive impairment. *Semergen*.

[8] Lamichhane B, Moukaddam N, Sabharwal A (2024). Mobile sensing-based depression severity assessment in participants with heterogeneous mental health conditions. *Scientific reports*.

[9] Liu L, Xu M, Marshall IJ, Wolfe CD, Wang Y, O’Connell MD (2023). Prevalence and natural history of depression after stroke: A systematic review and meta-analysis of observational studies. *PLoS medicine*.

[10] Kimball TN, Prapiadou S, Tack RWP, Yong-Qiang Tan B, Senff JR, Kourkoulis C (2025). Association of Leucocyte Telomere Length With Stroke, Dementia, and Late-Life Depression: The Role of Modifiable Risk Factors. *Neurology*.

[11] Jellinger KA (2023). The heterogeneity of late-life depression and its pathobiology: a brain network dysfunction disorder. *Journal of neural transmission (Vienna, Austria: 1996)*.

[12] Chen W, Shen Y, Song S, Li X (2025). Association of sleep duration and sleep disorders with post-stroke depression and all-cause and cardiovascular disease mortality in US stroke survivors: results from NHANES 2005-2018. *European journal of medical research*.

[13] Liu L, Li Z, Zhou H, Duan W, Huo X, Xu W (2023). Chinese Stroke Association guidelines for clinical management of ischaemic cerebrovascular diseases: executive summary and 2023 update. *Stroke and vascular neurology*.

[14] Campbell BCV, Khatri P (2020). Stroke. *Lancet (London, England)*.

[15] Hamilton M (1959). The assessment of anxiety states by rating. *The British journal of medical psychology*.

[16] Huang H, Lu M, Zhang P, Xiao L, Zhang W, Xu Y (2024). Association between malnutrition, depression, anxiety and fatigue after stroke in older adults: a cross-lagged panel analysis. *Aging clinical and experimental research*.

[17] Li W, Xiao WM, Chen YK, Qu JF, Liu YL, Fang XW (2019). Anxiety in Patients With Acute Ischemic Stroke: Risk Factors and Effects on Functional Status. *Frontiers in psychiatry*.

[18] HAMILTON M (1960). A rating scale for depression. *Journal of neurology, neurosurgery, and psychiatry*.

[19] Li J, Oakley LD, Brown RL, Li Y, Luo Y (2020). Properties of the Early Symptom Measurement of Post-Stroke Depression: Concurrent Criterion Validity and Cutoff Scores. *The journal of nursing research: JNR*.

[20] Brott T, Adams HP Jr, Olinger CP, Marler JR, Barsan WG, Biller J (1989). Measurements of acute cerebral infarction: a clinical examination scale. *Stroke*.

[21] Lee CF, Venketasubramanian N, Wong KS, Chen CL, Investigators CS (2016). Comparison Between the Original and Shortened Versions of the National Institutes of Health Stroke Scale in Ischemic Stroke Patients of Intermediate Severity. *Stroke*.

[22] Zhou Y, Zhou Y (2024). Non-adaptive cognitive emotion regulation mediates the relationship between disease uncertainty and acute stress disorder in patients with ischaemic stroke. *Frontiers in psychiatry*.

[23] Higueras-Fresnillo S, Herraiz-Adillo A, Ahlqvist VH, Oberg R, Lenander C, Wennberg P (2024). Associations of psychological factors with atherosclerosis and cardiovascular health in middle-age: the population-based Swedish CArdioPulmonary bioImage study (SCAPIS). *BMC public health*.

[24] Yin H, Gao C, Quan Z, Zhang Y (2023). The relationship between frailty, walking ability, and depression in elderly Chinese people. *Medicine*.

[25] Badwaik DG, Badwaik P (2021). Influence of Psychological Disorders on the Functional Outcomes in the Survivors of Ischemic Stroke. *Journal of stroke and cerebrovascular diseases: the official journal of National Stroke Association*.

[26] Garcia-Rudolph A, Sauri J, Garcia-Molina A, Cegarra B, Opisso E, Tormos JM (2022). The impact of coronavirus disease 2019 on emotional and behavioral stress of informal family caregivers of individuals with stroke or traumatic brain injury at chronic phase living in a Mediterranean setting. *Brain and behavior*.

[27] Cankaya S, Safa SS, Karakus A, Savcili M, Hanoglu L, Mardinoglu A (2025). Depression is an independent risk factor for stroke reccurence and cognitive impairment in stroke patients. *Translational psychiatry*.

[28] de Hartog-Keyzer JML, Pedersen SS, El Messaoudi S, Nijveldt R, Pop VJM (2022). Psychological distress is independently related to new coronary events at 8 years’ follow-up in elderly primary care patients with hypertension. *Journal of psychosomatic research*.

[29] Zhao X, Yuan X, Meng D, Liang H, Xiong Y, Li Y (2025). Prevalence and correlates of anxiety and depression among chronically ill older adults in Zunyi, China: a cross-sectional study. *Frontiers in psychology*.

[30] Qin L, Wang K, Jiang LP, Xiao Z, Luo S (2025). Correlation between anxiety-depression disorders and brain structural connectivity abnormalities after subarachnoid hemorrhage. *World journal of psychiatry*.

[31] Zhang J, Liu X, Zhang Z (2024). The trajectory and influencing factors of disability acceptance in patients with hypertensive intracerebral haemorrhage: a protocol for prospective longitudinal cohort study in Heyuan City, China. *BMJ open*.

[32] Jaiswal V, Ang SP, Suresh V, Joshi A, Halder A, Rajak K (2024). Association between baseline high-sensitive C-reactive protein, homocysteine levels, and post-stroke depression among stroke patients: a systematic review, meta-analysis, and meta-regression. *Current problems in cardiology*.

[33] Jin Y, Yu L, Li Y (2024). Paroxetine Effect on Nerve Growth Factor, Human Neurotrophin-4, Brain-Derived Neurotrophic Factor Levels in Post-stroke Depression. *Molecular neurobiology*.

[34] Tao Y, Wang W, Zhao J, Xu X, Ke J, Ke X (2023). The association between circulating Hcy and Lp-PLA2 levels and poststroke depression: A meta-analysis. *Medicine*.

